# Soil-Transmitted Helminths and *Schistosoma mansoni* Infections in Ethiopian Orthodox Church Students around Lake Tana, Northwest Ethiopia

**DOI:** 10.1371/journal.pone.0155915

**Published:** 2016-05-20

**Authors:** Aschalew Afework Bitew, Bayeh Abera, Walle Seyoum, Befekadu Endale, Tibebu Kiber, Girma Goshu, Addiss Admass

**Affiliations:** 1 ALKAN University College, Bahir Dar, Ethiopia; 2 Department of Microbiology, Immunology and Parasitology, College of Medicine and Health Sciences, Bahir Dar University, Bahir Dar, Ethiopia; UMASS Medical School, UNITED STATES

## Abstract

**Background:**

Soil-transmitted helminths (STH) and *Schistosoma mansoni* infections are the major neglected tropical diseases that result in serious consequences on health, education and nutrition in children in developing countries. The Ethiopian Orthodox church students, who are called *Yekolotemari* in Amharic, live in areas with poor sanitation and hygiene. Moreover, they are not included in the national STH control programs. Thus, STH and *S*. *mansoni* infections prevalence is unknown.

**Methods:**

A cross-sectional study was conducted on 384 students in June 2014 to determine STH and *S*. *mansoni* infections prevalence. Moreover, the knowledge of students about STH and *S*. *mansoni* was assessed. Data on knowledge and clinical symptoms were collected using structured questionnaires via face to face interview. Stool specimens were examined by formol-ether concentration method.

**Results:**

The overall prevalence of intestinal helminths infections was 85.9% (95% confidence interval (CI): 82.1–89%). STHs infections prevalence was 65.6% (95% CI: 60.7–70.2%). The prevalence of hookworm, *Ascaris lumbricoides* and *Trichuris trichiura* were 31.8% (95% CI: 27.3–36.6%), 29.4% (25–31%) and 3.1% (1.8–5.4%), respectively. On the other hand, *S*. *mansoni* prevalence was 14.3% (95% CI: 11.1–18.1%). Majority of students infected with *S*. *mansoni* had bloody stool with crud odds-ratio of 2.9 (95% CI: 1.5–5.5). Knowledge assessment showed that 50 (13%) and 18 (4.9%) of the respondents knew about transmission of STH and *S*. *mansoni*, respectively.

**Conclusions:**

The prevalence of STH and *S*. *mansoni* infections were high thus de-worming program should include the students of Ethiopian Orthodox churches. Furthermore, provision and use of sanitary facilities, health education for students to create awareness of parasitic infections and improved personal hygiene should be in place.

## Introduction

Soil-transmitted helminths (hookworm, *Ascaris lumbricoides* and *Trichuris trichiura*) and *S*. *mansoni* infections are the major neglected tropical diseases in sub-Saharan African including Ethiopia. These parasites are more prevalent in populations with low income, poor sanitation and overcrowded conditions. Moreover, limited access to clean water, bare footing, and irrigation practice are risk factors for STH and *S*. *mansoni* infections. According to World Health Organization estimates, more than 1.5 billion people are infected with soil-transmitted helminths worldwide [1].

Among soil-transmitted helminths, *A*. *lumbricoides*, *T*. *trichiura* and hookworm have particular public health importance because they have negative impact on the health, education, nutrition and economic development [2, 3] of the population. Therefore, STH and *S*. *mansoni* have been targeted for control and eradication [4]. The mapping of STH and *S*. *mansoni* are determined by climate, geography, poverty, environmental contamination and water bodies [2, 5].

Among sub-Saharan Africa, Ethiopia bears the 2^nd^, 3^rd^ and 4^th^ high burden of *A*. *lumbricoides*, hookworm and *T*. *trichiura*, respectively [6]. In Ethiopia, the prevalence and distribution of STH and *S*. *mansoni* varies because of differences in climate and humidity. Studies from various school aged children showed prevalence of 9.7 to 28.8% for hookworm, 9.9 to 12.7% for A. *lumbricoides* and 2.9–7.3% for *S*. *mansoni* [7, 8]. Compared to other sub-Saharan African countries, Ethiopia is the second country with high number of children in need of chemotherapy for de-worming helminths [9, 10]. Despite high burden of helminths, control of STH and *S*. *mansoni* in Ethiopia is at its infancy [11].

According to WHO report, schistosomiasis affects 240 million people worldwide. The majority of schistosomiasis cases are found in sub-Saharan Africa and more than 200 000 deaths occur per year [12]. *S*. *mansoni* is the most prevalent species in Ethiopia which causes intestinal schitosomiasis. In Ethiopia, 5.01 million people are estimated to be infected and 37.5 million are at risk for *S*. *mansoni* [13]. *S*. *mansoni* occurs mainly along streams, irrigation schemes and lakes.

The present study was conducted around Lake Tana area, which is situated in central highlands in northwestern Ethiopia. Its location and humid climate create conducive environment for transmission of STH and *S*. *mansoni*. Ethiopian Orthodox students are grouped in low socioeconomic classes and majority of students get local foods by begging different households in the name of St. Mery. The habit of bare foot, open defecation, swimming and bathing in river and drinking unsafe water are risk factors for acquiring STH and *S*. *mansoni*. Furthermore, Ethiopian Orthodox church students are not targeted so far in the national de-worming program. Therefore, students from the traditional schools may contribute for ongoing transmission of STH and *S*. *mansoni* in the community.

Although, data on STH and *S*. *mansoni* have been available in several Ethiopian school aged children little is known for students in the Ethiopian Orthodox churches. Therefore, this study was conducted to determine the prevalence of STH and *S*. *mansoni* infections in *Yekolotemari* around Lake Tana in northwestern Ethiopia. Moreover, the study assessed the knowledge of students about STH and *S*. *mansoni* transmission.

## Materials and Methods

### Study area and population

This study was conducted around Lake Tana located north of Bahir Dar City. Bahir Dar is located 565km to the northwest of Addis Ababa. The Lake Tana is famous for the ancient monasteries and churches. It has 20 monasteries and churches.

At the time of the study, there were 2500 male students (*Yekolotemari*). Ethiopian Orthodox church students (*Yekolotemari*) include any males who are willing to study all the educational structures. Studying the Ethiopian Orthodox church is a pre-requisite to transmit the role of the church and to become trained scholar for the church. They are grouped in low socio economic status; their livelihood is based on begging food items in the name of St. Mery by going from house to house in the community.

### Operational definition

*Yekolotemari* are traditional students who are from rural family of Ethiopia willing to study all the educational structures in Ethiopian Orthodox churches. They live in church compounds till they compete their education. Learning the Ethiopian Orthodox church is a pre-requisite to transmit the role of the churches and to become trained scholar.

### Study design and sample size

A cross-sectional study was conducted in June 2014. The sample size was determined at 95% level of confidence, with 5% margin of error and 50% prevalence of STH infection [14]. Hence, using single population proportion formula the sample size was estimated to be 384. From the twenty schools, eight churches were selected using simple random sampling technique. The number of students to be surveyed was calculated by dividing the sample size by the number of schools to be included in the study. Accordingly, from Azwa Mariam with n = 24, Ura Kidanimihiret n = 10, Selchen Mikaeal n = 7, Kibran Gebrael n = 19, Bata n = 83, Kiduse Georgis n = 41, Kiduse Mikael n = 103and Abuye with n = 97. The study participants were selected using simple random sampling method.

#### Data collection

Students’ knowledge about the transmission of intestinal parasites was collected via face to face interview using 6-question items. Clinical data were collected by experienced health officers. For stool examination, 3 grams of stool specimen was collected from each student using clean, dry, wide necked and leak proof plastic container. Upon delivery of the specimens, the containers were labeled and preserved with 10 ml of 10% normal saline solution. Immediately within 2 hours of collection, stool samples were transported to ALKAN University College Laboratory. The stool specimens were examined for intestinal parasites using formol-ether concentration technique by trained laboratory technologists.

#### Data analysis

Data were analyzed using the Statistical Package for Social Sciences version 20 software (IBM Corp. Released 2011. IBM SPSS Statistics for Windows, Armonk, NY: IBM Corp). Chi-square test and crude odds ratio were computed to test the association between categorical variables and parasitic infections. P-value <0.05 with 95% confidence interval (CI) was considered as statistically significant.

#### Mean score

Participants’ knowledge on STH and *S*. *mansoni* transmission was surveyed by 6 item-questions. The response alternatives for knowledge items were dichotomous. For knowledge assessment, each correct response was given a score of 1 while a wrong or doubtful response was scored as 0. Mean knowledge scores < 0.5 were considered as below the expected level of knowledge while average scores ≥0.5 deemed knowledgeable

#### Ethical considerations

Ethical clearance was obtained from research and ethics review committee of the ALKAN University College and Institutional Research Ethics Review Board of the Amhara Regional State Health Bureau, Research and Technology Transfer Core Process. Written consent was obtained from each participant. In this study, 257 participants whose age was below 18 years gave assent themselves in consultation to their guardians because children (minors) would not face any psychological and physical harmful procedures. Thus, the Institutional Research Ethics Review Board approved the consent form for children who participated in this research.

## Results

### Hygiene characteristics and knowledge of students

The mean age of the study participants was 20.8 years. More than 42% of students used to live in churches for two years and above. In this survey, 99% of the respondents did not get toilet facility thus used open field defecation. All of them take bath and clothes in Lake Tana. Sources of drinking water and hand washing practices are depicted in Table 1. In this survey, 50 (13.0%) of the respondents were knowledgeable on mode of transmission of STH. In contrast, only 18 (4.7%) had information about transmission of S. *mansoni*.

**Table 1:** Age and hygiene characteristics of students in Ethiopian Orthodox Church.

Variables	Number	Percentage
Age group		
Children (10–18yrs)	157	40.8
Adults (>18yrs)	227	59.2
Latrine use		
Use toilet	4	1.0
Open field	380	99.0
Hand washing after defection		
Yes	94	24.5
No	290	75.5
Drinking water sources		
Tap water	194	50.1
Well	77	20.1
Lake Tana	98	25.5
Lake Tana and well	15	3.9
Knowledge on STH		
Yes	50	13.0
No	334	87.0
Knowledge on *S*. *mansoni*		
Yes	18	4.7
No	366	95.3

### Prevalence of STH and *S*. *mansoni* infections

Overall, 330 (85.9%) of students at the selected churches were infected with intestinal helminths whereas the prevalence of STH infection was 65.6% (95% CI: 60.7–70.2%). Prevalence of *S*. *mansoni* infection was 14.3% (95% CI: 11.2–18.2%). Hookworm with 31.5% was the most prevalent STHs. Detail prevalence of each STH is shown in Table 2. Two or more intestinal helminths were found in 179 (46.6%) of the students. Thus, infection with single helminths was 150 (39%) whereas double and multiple infections (three and above) were 125 (32.6%) and 54 (14.0%), respectively.

**Table 2:** Prevalence of STH and *S*. *mansoni* in students in Ethiopian Orthodox Church.

Intestinal helminths	Age group								
	10–18 years (n = 157)			>18 years (n = 227)			Total		
	n	%	95% CI	n	%	95% CI	n	%	95% CI
Hookworm spp	42	26.7	20.4, 34.2	80	35.1	29.3, 41.6	122	31.8	27.3, 36.6
*A*. *lumbricoides*	50	31.8	25.1, 39.5	63	27.7	22.3, 33.9	113	29.4	25.1, 34.1
*T*.*trichiura*	6	3.8	1.7, 8.1	6	2.6	1.2, 5.6	12	3.1	1.8, 5.4
*E*.*vermicularis*	3	1.9	0.6, 5.4	3	1.3	0.4, 3.8	6	1.6	0.7, 3.3
*S*. *stercoralis*	3	1.9	0.6, 5.4	2	0.8	0,2, 3.1	5	1.3	0.6, 3.0
*S*.*mansoni*	25	16.0	11.0, 22.4	30	13.2	9.4, 18.2	55	14.3	11.2, 18.2
*H*.*nana*	8	5.1	2.6, 9.7	7	3.0	1.5, 6.2	15	3.9	2.3, 6.2
*Taenia* spp	1	0.6	0.1, 3.5	1	0.4	0.08, 2.4	2	0.5	0.4, 1.9
Overall prevalence	138	87.9	81.9, 92.1	192	84.6	79.3, 88.7	330	85.9	82.1, 89.1

Table 3 depicts the associations between clinical symptoms with STH and *S*. *mansoni*. Statistically significant association was observed between hookworm infection and abdominal pain (P = 0.021). Moreover, there was significant association between S. *mansoni* infection and appearance of bloody stool (P = 0.001).

**Table 3:** Association of clinical symptoms of STH and *S*. *mansonia* infections.

Helminths	Infection		COR (95% CI)	P value
**Hookworm**	Yes	No		
Abdominal pain	97	181	1.00	0.021
	25	81	1.74 (0.01, 2.99)	
Bloody stool	23	65	1.00	0.122
	99	184	0.66 (0.37, 1.16)	
***A*. *lumbriciodes***	Yes	No		
Abdominal pain	86	192	1.3 (0.77, 2.24)	0.17
	77	29	1.00	
***S*. *mansoni***	Yes	No		
Abdominal pain	39	139	1.00	0.45
	16	90	0.92 (0.47, 1.8)	
Mucoid stool	20	56	2.58 (1.32, 5.02)	0.34
	35	253	1.00	
Bloody stool	23	65	2.9 (1.5, 5.5)	0.001
	32	241	1.00	

Key: COR: crude odds-ratio.

## Discussion

This study revealed the first prevalence of STH and *S*. *mansoni* infections in Ethiopian Orthodox church students. As expected, the prevalence of intestinal helminths was very high. The prevalence of STH and *S*. *mansoni* infections in this study were significantly higher than the prevalence of helminths reported from school children in the same geographical areas in northwestern Ethiopia [6, 8, 15]. The prevalence of STH in Yekolotemari was above 50%. Therefore, according to WHO guideline, bi-annual chemotherapy is required to reduce STHS’ egg burden in the community [1].

High prevalence of STHs found in this study might be attributable to open field defecation, poor hand washing practice and low level of awareness about STHs transmission. Furthermore, students in the surveyed Orthodox churches are not included in school-deworming program. Therefore, unless intervention is done, students in Ethiopian Orthodox churches may remain sources for helminths transmission thereby impacting STH control. Hence interventions including mass de-worming and improving sanitation and personal hygiene should be in place.

The prevalence of hookworm infections in this study was higher than the national prevalence of hookworm (16%) [16]. It was also higher than prevalence of hookworm in school children in Gondar Ethiopia [8, 17]. Among STHs, the prevalence of hookworm infection was higher in young adults than children. Likewise, in Brazil high prevalence of hookworm was noted in adults [18]. In contrast, other STHs were higher in young children than adults.

The prevalence of *A*. *lumbricoides* in this study was higher than other studies in different part of the country in modern school children in Ethiopia [7, 8, 19, 20]. The prevalence of *T*. *trichiura* in this study was higher than school children in same study area [7]. However, prevalence of *T*. *trichiura* was comparable with other studies in south Gondar, Ethiopia [8, 20]. The prevalence of S. *mansoni* in this study was higher than school based reports [7, 8]. This showed that students in Ethiopian Orthodox churches were the highest risk groups in the community and serve as sources of *S*. *mansoni* transmission.

Statistical significant association was observed between clinical symptoms in hookworm and *S*. *mansoni* infected students. Significant proportions of hookworm infected students had abdominal cramp than non-hookworm infected students with COR 1.4. Majority of students infected with *S*. *mansoni* had bloody stool with COR 2.9. Similarly, hookworm and *S*. *mansoni* infected children had lower hematocrit values [7]. However, other studies did not show any significant association between the helminths and clinical symptoms [14, 16]. Clinical manifestations in children with STH rely on the load of worms in the intestine. The main limitation of this study was that the intensity of parasitic load was not measured. The exact risk factors for acquisition of STH and *S*. *mansoni* in *Yekolotemaria* were not determined due to the cross-sectional study design effect.

## Conclusion

This study has shown high prevalence of STHs and *S*. *mansoni* infection in Ethiopian Orthodox church students (*yekolotemaria*) around Lake Tana in northwestern Ethiopia. All students used open field defecation near Tana Lake. Furthermore, majority of students had knowledge gap on STH and *S*. *mansoni* transmission. Thus, students in Ethiopian Orthodox churches are deemed pockets for ongoing transmission of STH and *S*. *mansoni* in northwest Ethiopia.

### Recommendation

We recommend that de-worming programs should include students in the Ethiopian Orthodox churches.

The de-worming program should also be supplemented with other measures such as provision of sanitary facilities, health education for students to create awareness of parasitic infections and improved personal hygiene.
